# A Free-Standing Sulfur/Nitrogen-Doped Carbon Nanotube Electrode for High-Performance Lithium/Sulfur Batteries

**DOI:** 10.1186/s11671-015-1152-4

**Published:** 2015-11-19

**Authors:** Yan Zhao, Fuxing Yin, Yongguang Zhang, Chengwei Zhang, Almagul Mentbayeva, Nurzhan Umirov, Hongxian Xie, Zhumabay Bakenov

**Affiliations:** Research Institute for Energy Equipment Materials, Hebei University of Technology, Tianjin, 300130 China; Tianjin Key Laboratory of Laminating Fabrication and Interface Control Technology for Advanced Materials, Hebei University of Technology, Tianjin, 300130 China; Institute of Batteries LLC, 53 Kabanbay Batyr Avenue, Astana, 010000 Kazakhstan; PI Nazarbayev University Research and Innovation System, Nazarbayev University, 53 Kabanbay Batyr Avenue, Astana, 010000 Kazakhstan

**Keywords:** Lithium/sulfur battery, Sulfur/nitrogen-doped carbon nanotube composite cathode, Free-standing electrode, 82.47.Aa, 82.45.Fk

## Abstract

A free-standing sulfur/nitrogen-doped carbon nanotube (S/N-CNT) composite prepared via a simple solution method was first studied as a cathode material for lithium/sulfur batteries. By taking advantage of the self-weaving behavior of N-CNT, binders and current collectors are rendered unnecessary in the cathode, thereby simplifying its manufacturing and increasing the sulfur weight ratio in the electrode. Transmission electronic microscopy showed the formation of a highly developed core-shell tubular structure consisting of S/N-CNT composite with uniform sulfur coating on the surface of N-CNT. As a core in the composite, the N-CNT with N functionalization provides a highly conductive and mechanically flexible framework, enhancing the electronic conductivity and consequently the rate capability of the material.

## Background

Lithium/sulfur (Li/S) batteries possess great potential as advanced rechargeable batteries for electric vehicles (EVs) and hybrid electric vehicles (HEVs) due to their large theoretical capacity at 1672 mAh g^−1^ and high theoretical energy density of 2600 Wh kg^−1^ [1, 2]. Furthermore, as a cathode material, sulfur has the advantages of natural abundance, low cost, and environmental friendliness [3]. However, the commercialization of Li/S batteries faces several challenges related to insulating the nature of sulfur, solubility of polysulfides as discharge products in the electrolyte, and volume change of sulfur cathode during lithiation/delithiation [4, 5].

To circumvent the problem, various efforts have been made and various types of conductive carbon materials and conductive polymers have been used to composite with sulfur in order to enhance the electric conductivity of the sulfur composite and hinder the dissolution of the polysulfides into the electrolyte [6–14]. Among them, carbon nanotubes (CNTs), with their high electrical conductivity and a unique tubular structure, are widely used as a flexible matrix to form composite cathodes for Li/S batteries [11–13]. Notably, it was reported that nitrogen-doped carbon nanotubes (N-CNTs) have a significantly improved electronic conductivity due to the nitrogen atoms providing additional free electrons for the conduction band [15, 16]. Furthermore, Sun et al. demonstrated that compositing the nitrogen-doped mesoporous carbon with sulfur leads to easy and enhanced sulfur reduction activities [14].

In this work, for the first time, we introduced the binder-free sulfur/nitrogen-doped carbon nanotube (S/N-CNT) composite prepared by a simple solution mixing method as a cathode material for Li/S batteries. It was demonstrated that utilization of N-CNT has led to the high electrochemical performance, suggesting the great potential of N-CNT as a cathode additive for high-performance lithium/sulfur batteries.

## Methods

**Fig. 1:**
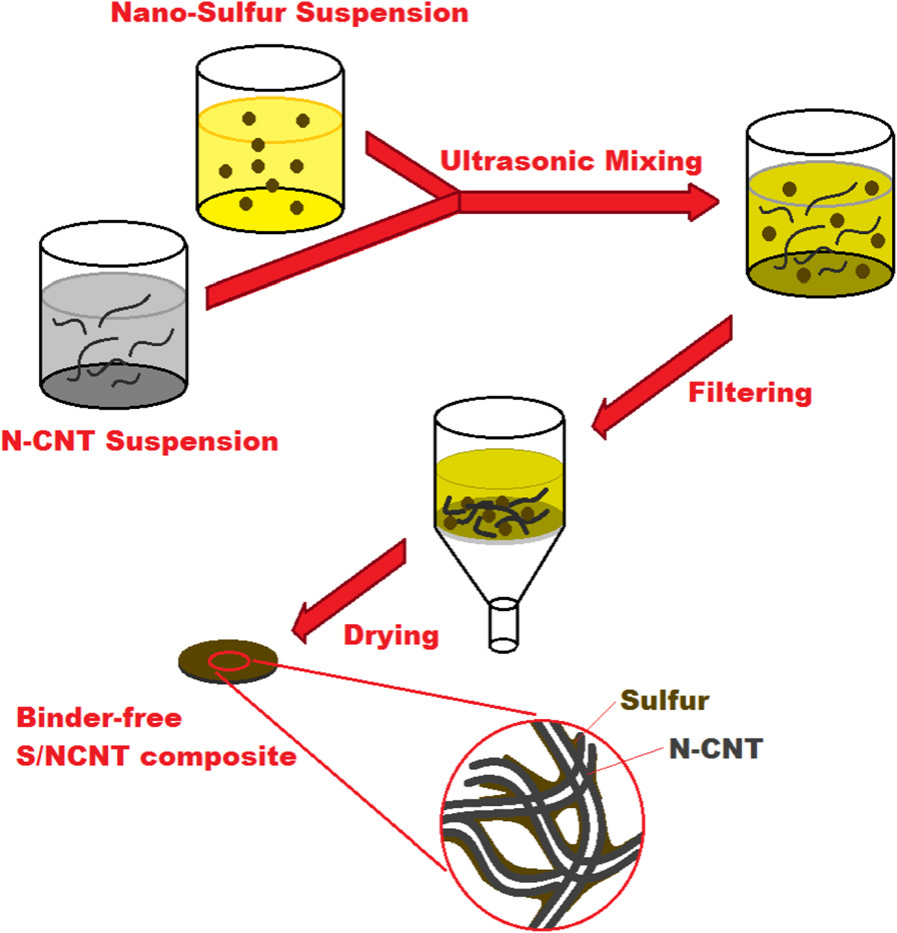
Schematic diagrams of the synthesis of S/N-CNT composite

The S/N-CNT composite preparation is schematically presented in Fig. 1; 0.15 g nitrogen-doped carbon nanotubes (US Research Nanomaterials Inc) were first dispersed in 40 mL deionized water by sonication (Fisher Scientific, FB120) at room temperature for 2 h. The resulting N-CNT suspension and 3 g aqueous suspension of nano-sulfur (US Research Nanomaterials Inc, 10 wt%) were simply mixed and ultrasonicated for 2 h. The S/N-CNT samples were vacuum-filtered and washed three times with deionized water and ethanol. The binder-free S/N-CNT composite was obtained by further drying the sample in a vacuum oven at 60 °C overnight to remove the solvent.

The surface morphology and microstructure of the composite were examined by field emission scanning electron microscopy (SEM, JSM-6490, JEOL) and high-resolution transmission electron microscopy (HRTEM, JEM-2800, JEOL) with energy dispersive spectroscopy (EDX) mapping. The S content in the S/N-CNT composite was determined using chemical analysis (CHNS, Vario Micro Cube, Elementar). The electrochemical performance of the S/N-CNT composite cathode materials was investigated using coin-type cells (CR2032). The cell was composed of lithium metal anode and S/N-CNT cathode separated by a microporous polypropylene separator soaked in 1 M lithium bis (trifluoromethanesulfonate) (Aldrich) in tetraethyleneglycol dimethyl ether (Aldrich) electrolyte. The resulting cathode film was used to prepare the cathodes by punching circular disks with 1 cm in diameter. The coin cells were assembled in an Ar (99.9995 %)-filled glove box (MBraun) and tested galvanostatically on a multichannel battery tester (BTS-5V5mA, Neware). The cyclic voltammetry tests were performed using VMP3 potentiostat/galvanostat (Bio-Logic Science Instrument Co.). Applied currents and specific capacities were calculated on the basis of the weight of S in each cathode.

## Results and Discussion

**Fig. 2:**
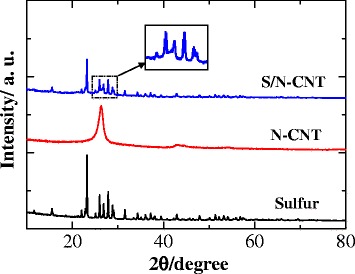
XRD patterns of elemental sulfur, N-CNT, and S/N-CNT composite

The structure of the S/N-CNT composite was studied by using X-ray powder diffraction (XRD). Figure 2 presents the XRD results for S, N-CNT, and the resulting composite. The XRD patterns of sulfur exhibit the characteristic features of *Fddd* orthorhombic structure. The N-CNT displays a strong diffraction peak at 26° and a weak one at 43°, corresponding to the (0 0 2) and (1 0 0) planes [15, 16]. The characteristic bands of the S/N-CNT composite are consistent with that of the elemental sulfur but with reduced intensity, which indicate the well-dispersed character of nanoscopic sulfur in the composite structure, as evidenced further by electron microscopy analysis as well. Furthermore, one can observe in the characteristic bands of the S/N-CNT composite that the base line of XRD peaks around 26° is slightly raised. This was ascribed to the dispersed N-CNT and indicates that a homogeneous mixture of S/N-CNT was obtained by a simple solution mixing method [11].

The chemical analysis of the composite confirmed a high sulfur content of 61 wt%, which was possible due to the formation of self-standing film and avoiding the needs of using a polymer binder.

**Fig. 3:**
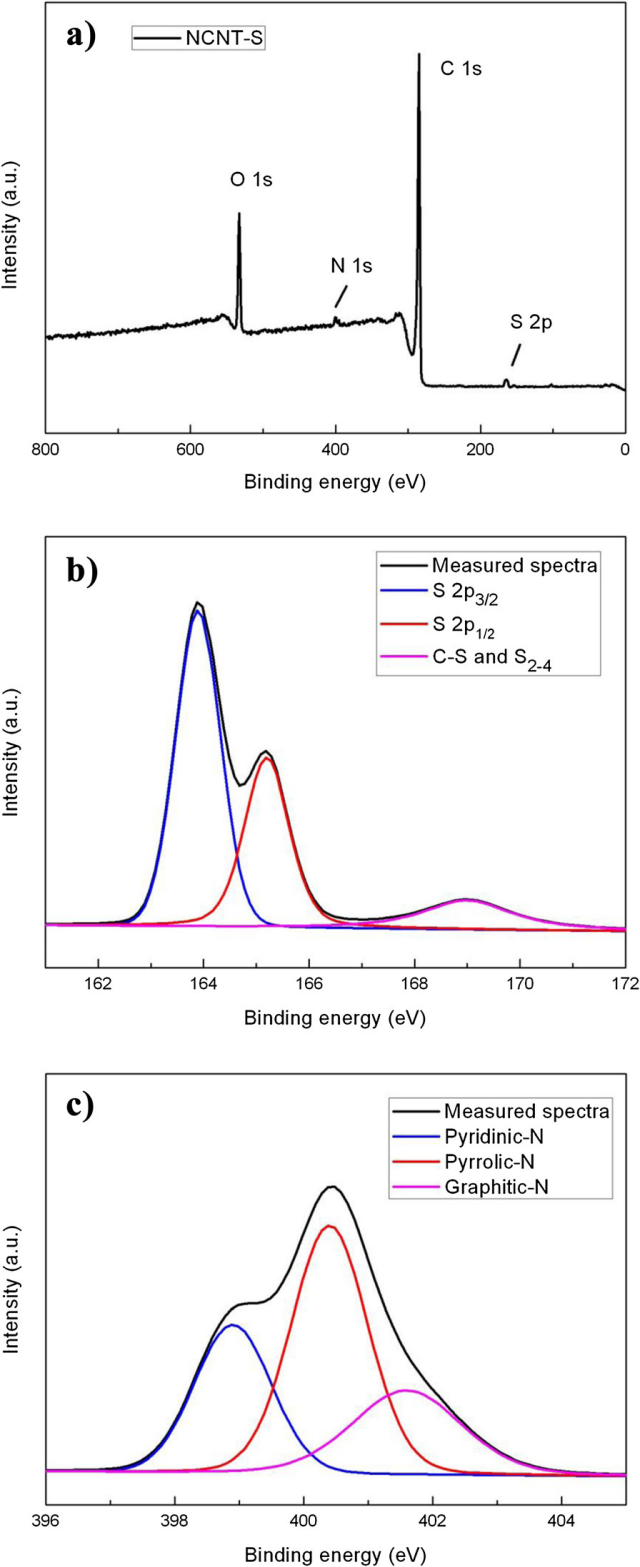
(**a**) XPS survey spectra of S/N-CNT; high-resolution XPS spectra of (**b**) S 2p and (**c**) N 1s in the S/N-CNT

XPS characterizations were performed to further analyze the chemical composition and surface properties of the S/N-CNT composite. The survey spectra in Fig. 3a prove that four peaks at 164, 290, 401, and 530 eV are attributed to S2p, C1s, N1s, and O1s, respectively. The S2p peaks (Fig. 3b) can be divided into two components including S2p3/2 peak (163.7 eV) and S2p1/2 peak (164.9 eV), and another weak broad peak located between 167.5 and 170.5 eV can be attributed to the interaction between sulfur and CNT or surface oxidation of sulfur [17]. The high-resolution N1s peaks (Fig. 3c) can be deconvoluted into three components including pyridinic-N (398.7 eV), pyrrolic-N (400.4 eV), and graphitic-N (401.9 eV), respectively. The nitrogen doping can enhance the surface absorption to soluble polysulfides and improve the electronic conductivity of carbon matrixes, thereby improving the electrochemical activity and the utilization rate of sulfur [18].

**Fig. 4:**
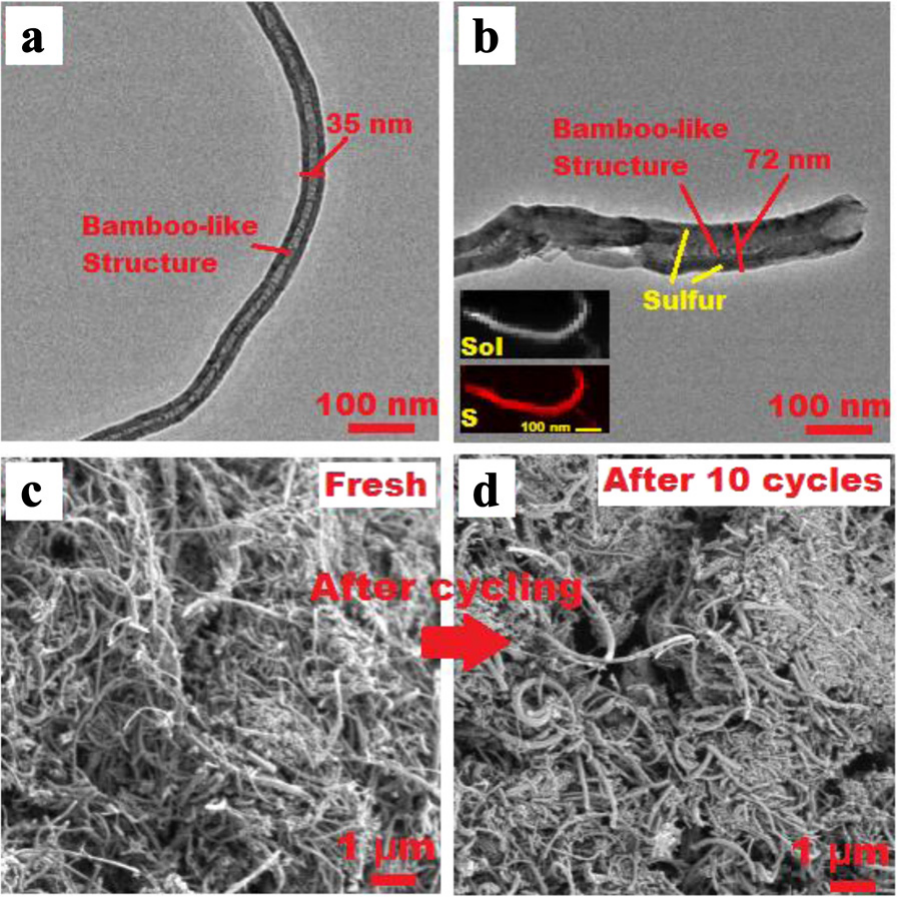
**a**, **b** TEM images of N-CNT and S/N-CNT composite. *Inset* EDS mapping showing distribution of S in the S/N-CNT composite. **c**, **d** SEM images of S/N-CNT composite before and after discharge/charge cycles

The structure of the N-CNT and S/N-CNT composite is imaged by TEM as depicted in Fig. 4a, b. One can see from Fig. 4a that the N-CNT possesses a typically bamboo-like structure, demonstrating that nitrogen was successfully introduced into the carbon network [19]. During the mixing process of the N-CNT and nano-sulfur aqueous suspension, the surface of N-CNT was mostly occupied by active sulfur for lithium ion storage. Thus, the diameter of N-CNT increases from 35 to 72 nm. This is in good agreement with the TEM-EDS mapping, which reveals that sulfur homogenously coats N-CNT. As a core in the composite, the N-CNT can provide a high electronic conductivity and robust framework [20]. Besides, the network-like structure of the S/N-CNT composite favors the penetration of the electrolyte into the cathode [10]. To demonstrate the integrity of the structure of the S/N-CNT composite, the comparative SEM of fresh and cycled S/N-CNT composite is conducted. One can see from Fig. 4c, d that the S/N-CNT composite does not change remarkably upon cycle and remains its nanostructure. This analysis of micrographs obtained for the fresh and cycled cathode confirms that both morphology and structure were retained after the cycling, which leads to an excellent cyclic stability.

**Fig. 5:**
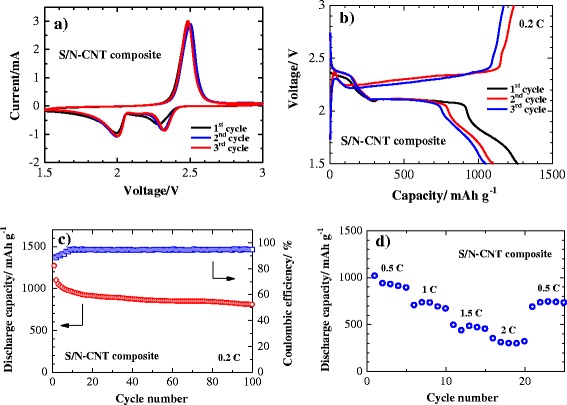
Electrochemical performance of a lithium cell with the S/N-CNT composite cathode. **a** Cyclic voltammograms at 0.1 mV s^−1^ scan rate. **b** Discharge/charge profiles at 0.2 C. **c** Cycling performance of the S/N-CNT composite cathode at 0.2 C. **d** Rate capability of the S/N-CNT composite cathode

The initial three cyclic voltammetry (CV) curves of a Li/S cell with the S/N-CNT composite cathode are shown in Fig. 5a. The CV data evidence two redox processes in the system which agrees well with the literature data [21] and could be attributed to the transition of S to polysulfides (Li_2_S_8_, Li_2_S_6_, Li_2_S_4_) and their further transformation to lithium sulfide Li_2_S, respectively. In the initial cycles, the activation process associated with the formation of SEI film and the transport of the electrolyte into the porous S/N-CNT composite result in an anodic peak at slightly lower potential. After this activation, the heights of the main peaks remain at a similar level, indicating good reversibility of the redox processes [22, 23].

The electrochemical performance of the S/N-CNT composite as a cathode material in Li/S batteries was further investigated by galvanostatic discharge/charge tests, and the results are displayed in Fig. 5b. The first plateau at about 2.4 V is related to the formation of higher-order lithium polysulfides (Li_2_S_n_, *n* ≥ 4), which are soluble in the liquid electrolyte. The following electrochemical transition of these polysulfides into lithium sulfide Li_2_S is associated to a prolonged plateau around 2.0 V, which are well-corresponded with the CV data. Figure 5c presents the cycling performance of the S/N-CNT composite at 0.2 C. The S/N-CNT composite exhibited a stable cycling behavior with small capacity loss even after 100 cycles. A reversible capacity of 1098 mAh g^−1^ was obtained by the S/N-CNT composite in the second cycle, and the cell retained about 73.5 % of its initial reversible discharge capacity after 100 cycles; the coulombic efficiency was maintained above 93 %. Based on the above phenomenon, we can conclude that the N-CNT could diminish the polysulfide dissolution in a physical and chemical way, thereby stabilize the capacity significantly. Furthermore, the free-standing S/N-CNT composite film possesses a robust and flexible structure, which could accommodate the solubilization/precipitation of sulfur during the cycles [24].

The rate capability results, as depicted in Fig. 5d, reveal excellent performance of the S/N-CNT composite at various current densities from 0.5 to 2 C. At the initial cycle at 0.5 C current, the composite achieves a discharge capacity of 1016 mAh g^−1^. There is a gradual capacity reduction with the increase in the current rate, although 298 mAh g^−1^ reversible capacity was sustained even at 2 C rate. More importantly, the composite regained the most of its reversible capacity (742 mAh g^−1^) when the discharge rate was modulated back to 0.5 C, which shows a high abuse tolerance of the S/N-CNT composite. This superb rate performance can be attributed to the excellent high-rate discharge capability of the composite sulfur cathode due to the good electrical conductivity of N-CNT and existence of good lithium ion transport path in the composite structure.

**Fig. 6:**
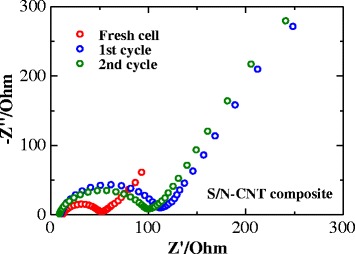
AC impedance spectroscopy data of a lithium cell with the S/N-CNT composite cathode

The EIS (Electrochemical Impedance Spectroscopy) measurements of the lithium half-cell with the S/N-CNT composite cathode were carried out before and after the galvanostatic discharge/charge. Figure 6 presents the trends in the EIS spectra change for these systems upon cycling. It can be seen that the impedance spectra contain a high-to-medium frequency semicircle and a low-frequency straight line. While the low-frequency straight line is attributed to the “Warburg impedance” resembling the solid-state diffusion of Li ions within the solid active mass [21], the compressed semicircle is mainly attributed to the charge-transfer (CT) impedance at the electrode/electrolyte interface. The CT impedance of the S/N-CNT composite cathode slightly increases and stabilizes after the initial cycles, which could be due to the formation of an interfacial layer, which protects the composite cathode from further dissolution of sulfur but has slightly lower conductivity than the composite itself. The cyclability data presented above agrees with these suggestions: the cyclability of the composite cathode stabilizes after a few initial cycles.

To understand the enhancement of the electrochemical performance from the nitrogen doping, further comparison between the S/CNT and S/N-CNT composite are carried out as shown in Table 1. It is worth noting that the as-prepared S/N-CNT composite in our work exhibited superior electrochemical performance in comparison with those previously reported results [25–29]. The result indicates that N-CNT with N functionalization could provide a highly conductive and mechanically flexible framework, which explains the reason why the S/N-CNT composite shows enhanced performance. Therefore, we could conclude that adopting nitrogen-doping carbon-based materials to composite with sulfur is proved to be a promising way to develop high-performance cathode materials for Li/S batteries.

**Table 1:** Literature comparison between the electrochemical performances of S/CNT and S/N-CNT composite cathodes for Li/S batteries

Materials	1st discharge capacity (mAh g^−1^)	*n*th cycle reversible capacity (mAh g^−1^)	Current density	Reference
Sulfur-porous carbon nanotubes	895	625 (100th)	0.1 C	[25]
Sulfur-multiwalled carbon nanotubes	1254	716 (40th)	0.2 C	[26]
Aligned carbon nanotube/sulfur	1097.5	~600 (85th)	0.1 C	[27]
Multiwalled carbon nanotubes-sulfur	741	592 (50th)	200 mA g^−1^	[28]
Sulfur-MWNTs	1274	637 (100th)	0.1 C	[29]
Sulfur/N-CNT composite	1267	807 (100th)	0.2 C	This study

## Conclusions

In this work, the free-standing S/N-CNT composite was prepared by simply mixing nanosized sulfur particle suspension and nitrogen-doped carbon nanotube suspension followed by vacuum filtration. In the composite, N-CNT serves as carbon skeleton for the S/N-CNT composite, forming a stable interconnected network structure. The S/N-CNT composite cathode exhibits good cyclability and rate capability in rechargeable lithium/sulfur battery. Moreover, the formation of the free-standing and flexible film of the S/N-CNT composite allowed for increasing the active cathode material content (S), and it makes this approach to be one of the possible ways to prepare flexible and/or bendable next-generation Li/S batteries.
